# Unmasking the Influence of Viral Micro‐RNAs on Host Immunity

**DOI:** 10.1111/imcb.70164

**Published:** 2026-09-22

**Authors:** Riley J. K. Easton, Bernadette M. Saunders

**Affiliations:** ^1^ School of Life Sciences, Faculty of Science University of Technology Sydney Ultimo Australia; ^2^ Tuberculosis Group, Infectious Diseases and Immunity Centenary Institute Camperdown Australia

## Abstract

Micro‐RNAs (miRNAs) are key regulators of human immunity, controlling critical cellular programmes including proliferation, differentiation, apoptosis, and immune activation to shape the host response to infection. Multiple human‐infecting viruses encode their own miRNA (v‐miRNA), enabling them to co‐opt host gene regulatory machinery to subvert immunity, establish persistence and evade immune‐mediated killing. This review synthesizes the current evidence on viral miRNAs and their roles in host–virus interactions, with an explicit focus on immune regulation. We examine how different viruses use v‐miRNAs to modulate core immune functions, including antigen presentation, cytokine signaling, NK and CD8 T‐cell activation, and apoptosis. We also examine how v‐miRNAs manipulate viral gene expression to aid immune evasion and promote persistence. Particular emphasis is placed on experimentally validated immune targets and their clinical consequences. Collectively, these examples illustrate the convergent evolution of v‐miRNA‐mediated immune evasion across diverse viral families. Understanding this class of molecules will provide insight into critical immune evasion strategies employed by clinically important viruses and may identify novel targets for diagnostic and antiviral therapeutics, including antagomir‐based and RNA silencing approaches.

## Graphical abstract

Viral micro‐RNAs are increasingly recognized as key modulators of host–virus interactions. They influence multiple immune functions, including antigen presentation, T‐cell activation, and cytokine signaling, while also tuning viral gene expression to promote persistence. This review highlights these understudied molecules and the additional layers of immune regulation they uncover.

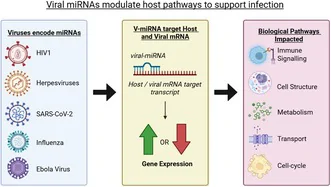

## 1 Viral Micro‐RNA: Challenging Genetics and Linguistics

MicroRNA are small, noncoding RNA molecules that regulate transcription by enhancing or repressing target mRNA expression. Since their discovery in humans in 2001, miRNA have been recognized as indispensable regulators of virtually every aspect of cellular and immune processes, a contribution underscored by the 2024 Nobel Prize in Physiology or Medicine. miRbase, a publicly available repository for micro‐RNA sequences, now lists 38 589 hairpin micro‐RNA precursors and 48 860 mature micro‐RNAs from 271 species [1], 148 animal and 82 plant kingdoms, including 34 viruses. These 34 viruses encode a reported 569 mature viral miRNAs.

Virus‐encoded miRNAs (v‐miRNAs) were first identified in Epstein–Barr virus (EBV) infected cells in 2004 [2]. For the first time, virologists demonstrated that viruses can directly regulate host gene expression at the posttranscriptional level, using the same small RNA machinery that the host deploys in coordinating the immune response against the virus. Since this discovery, functional v‐miRNAs have been identified in numerous viral strains, predominantly Class I double‐stranded DNA viruses. Beyond dsDNA viruses, certain RNA viruses that access the nucleus, such as retroviruses, are also proposed to produce v‐miRNAs, though the biogenesis and the biological and functional significance of these molecules remain the subject of intense study [3, 4]. There are several examples of putative v‐miRNAs produced by RNA viruses, which bypass the canonical host‐miRNA nuclear production machinery entirely (Figure 1) [5, 6, 7]. Dengue virus [8], Ebola [9], Enterovirus [10], Hepatitis A and C [5, 6], Poliovirus [6], Vesicular stomatitis virus [6], and West Nile virus [6] have all been found to produce virus‐derived‐small RNA. In silico‐predicative tools indicate that these molecules have an miRNA‐like structure that may have a biological effect on human mRNA. While some experimentally validated v‐miRNAs and their targets have been demonstrated for viruses like Ebola, experimental validation of svRNA's from other RNA viruses requires further investigation.

**FIGURE 1:**
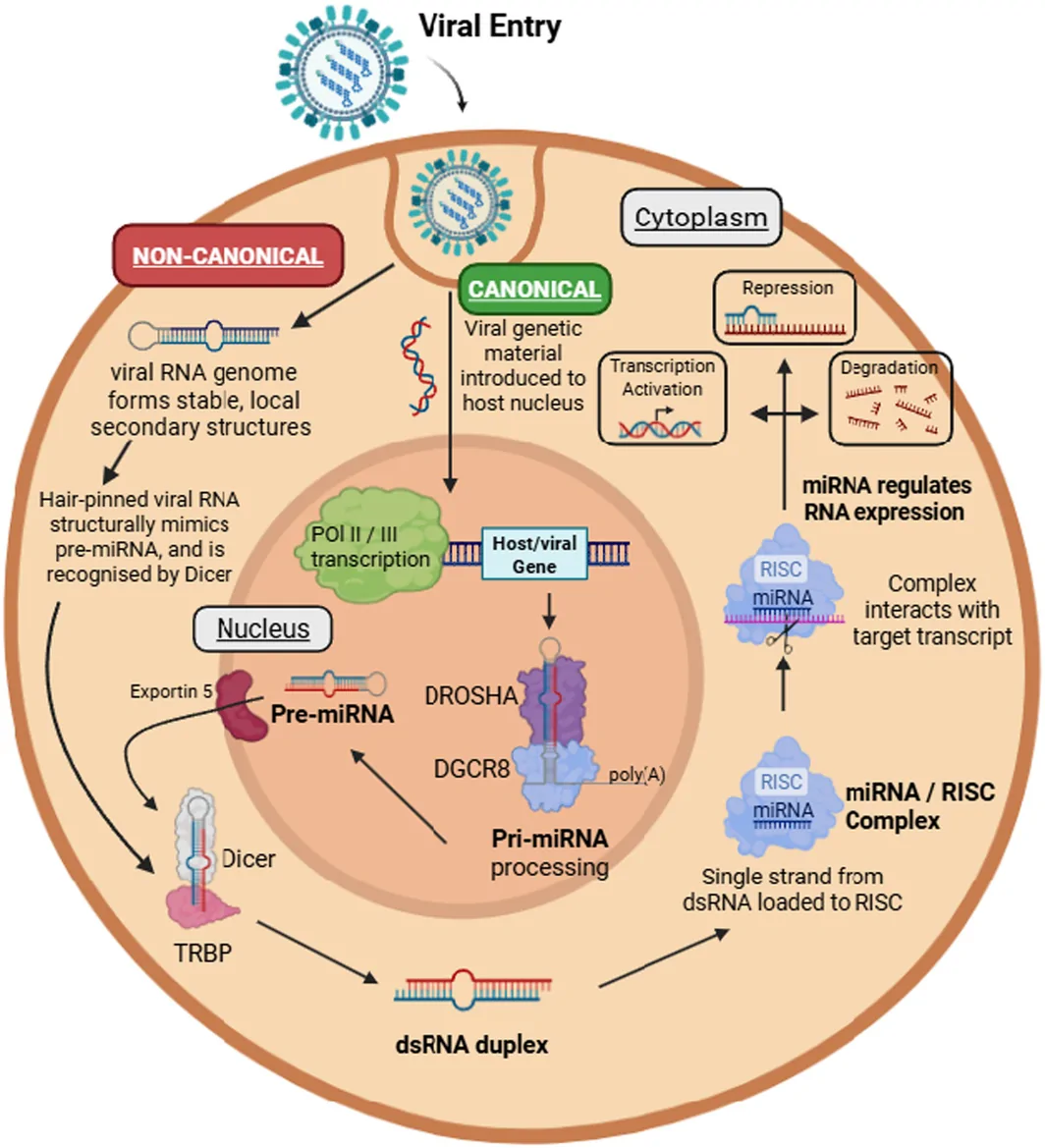
Viruses can exploit host miRNA biogenesis pathways through both canonical and noncanonical mechanisms. Canonical pathway: Viral genetic material entering the nucleus is transcribed by RNA polymerase II/III into pri‐miRNAs. These are processed by DROSHA/DGCR8 into pre‐miRNAs, exported via Exportin‐5, and cleaved by DICER/TRBP in the cytoplasm to form miRNA duplexes. One strand is loaded into AGO2‐RISC, guiding repression or degradation of target transcripts. Noncanonical pathway: Cytoplasmic RNA viruses bypass nuclear processing. Structured regions of viral RNA mimic pre‐miRNAs and are directly recognized by DICER/TRBP, producing miRNA‐like duplexes without DROSHA or Exportin‐5 involvement. These viral miRNAs still integrate into RISC to modulate gene expression.

While multiple repositories catalogue miRNA‐encoding organisms, miRbase v22 remains the most comprehensive reference database [1]. Additionally, several recent publications have identified viral miRNAs that are yet to be added to these repositories. Indeed, what actually constitutes a viral miRNA, and therefore should be in these repositories, is the subject of ongoing debate. Terminology in the literature reflects this uncertainty, with researchers using v‐miRNA, small‐viral RNA, virus‐derived small RNA, and miRNA‐like small RNA for what may be functionally equivalent molecules. This lack of centralized standardization and uniform naming conventions complicates the fundamental question: What structurally and functionally defines a viral‐microRNA? This question has been discussed in the past [11], and no doubt will be discussed more going forward, but generally, researchers distinguish “true v‐miRNAs” by looking for evidence of canonical biogenesis, structural and processing features, argonaute loading patterns, and functional targeting.

However, this search for a “true v‐miRNA” definition ignores the rapid growth of this area of research. Growth that is continually challenging our previous “true” definitions, such was the discovery of RNA‐virus v‐miRNA. Because of this focus on nomenclature, there is little synthesis regarding the immunological importance of this field of research. As alternative pathways for generating small nonhost miRNA or miRNA‐like RNA are discovered, current definitions no longer fit. While further discussions are required to define the parameters of these viral‐encoded miRNA or miRNA‐like small RNA, it is clear that these molecules can significantly impact human immunity. For this review, we do not throw any more hats into the ring or try to challenge different studies' decisions. Instead, we take their work at face value and refer to the RNAs as described in the original text, respecting the authors' preferences and applied methodologies. We will focus on how these molecules shape host antiviral immunity, exploring the immune pathways and effector mechanisms they target and reviewing emerging evidence for functional v‐miRNA across key human‐infecting viruses. We also discuss how viruses and their v‐miRNA have evolved alongside humans and our miRNA to overcome and exploit immunological features and systems.

### 1.1 Identifying and Validating v‐miRNA Targets

Viral miRNAs can regulate the expression of both viral genes and transcripts of the host cell they infect. As with human miRNA, in silico tools can be used to predict potential v‐miRNA targets [12]. Due to their small viral genomes, predicting viral targets is generally more straightforward than predicting host targets. For example, dsDNA viruses like herpesviruses possess genomes up to 240 kbp that encode 80–200 protein‐coding genes; the human genome encodes thousands. Interestingly, research into herpesvirus v‐miRNA targeting indicates that relatively few viral genes are directly regulated by v‐miRNA; instead, these viral molecules appear to preferentially target host genes to modify the cellular environment [13].

Predicting the host targets of v‐miRNA is complicated, not only by the sheer number of protein‐coding genes in humans but also by factors such as v‐miRNA localization, host immune responses, co‐infection factors, and the clinical stage of infection [3]. Context‐dependent targeting is another complication, as many v‐miRNA function only in specific cell types and at specific phases of the viral life cycle, such as latent or lytic replication [14, 15].

Conversely, some v‐miRNAs have been reported to regulate multiple host pathways through interactions with numerous target transcripts. HCMV‐miR‐Ul112‐1, for example, suppresses NK cell‐mediated killing of infected cells and has been implicated in the regulation of apoptosis and pro‐inflammatory cytokine release [16]. Because individual v‐miRNAs can influence multiple targets, their effects may influence infection dynamics across several biological processes and vary according to cell types, tissue environment, and stage of infection. This capacity for broad regulatory activity highlights how relatively small viral genomes can use v‐miRNAs to fine‐tune host responses and promote viral persistence.

The ability of v‐miRNAs to influence multiple cellular pathways is not restricted to broad target recognition alone. Some viral miRNAs also achieve this by mimicking host miRNAs. A prominent example is KSHV‐miR‐K12‐11, which shares 100% sequence identity with the human miRNA miR‐155. Based on in silico predictions, reporter assays and expression profiling, the two miRNAs were found to repress a common set of 66 target genes that may contribute to the development of primary effusion lymphoma [17]. Determining whether a host or viral miRNA is responsible for a particular phenotypic change remains challenging. The outcome depends not only on the response of infected host cells but also on competition with endogenous host miRNAs that may be responding to the infection or just maintaining cellular homeostasis. Furthermore, because a single v‐miRNA can regulate multiple host genes, the downstream effects may extend beyond its immediate targets. Experimentally, differentiating between direct and indirect effects of v‐miRNA can be difficult and requires careful experimental validation using a range of techniques [12].

Figure 2 summarizes the validated targets of several v‐miRNAs and highlights specific aspects of host immunity that they interact with. The validated targets of v‐miRNA were run through g‐profiler to identify biological pathways related to the input list of genes. The resulting biological pathways were then classified into one of five functional categories: Immune Signaling, Cellular Structure, Metabolic Processes, Transport Dynamics, and Cell Cycle Interactions.

**FIGURE 2:**
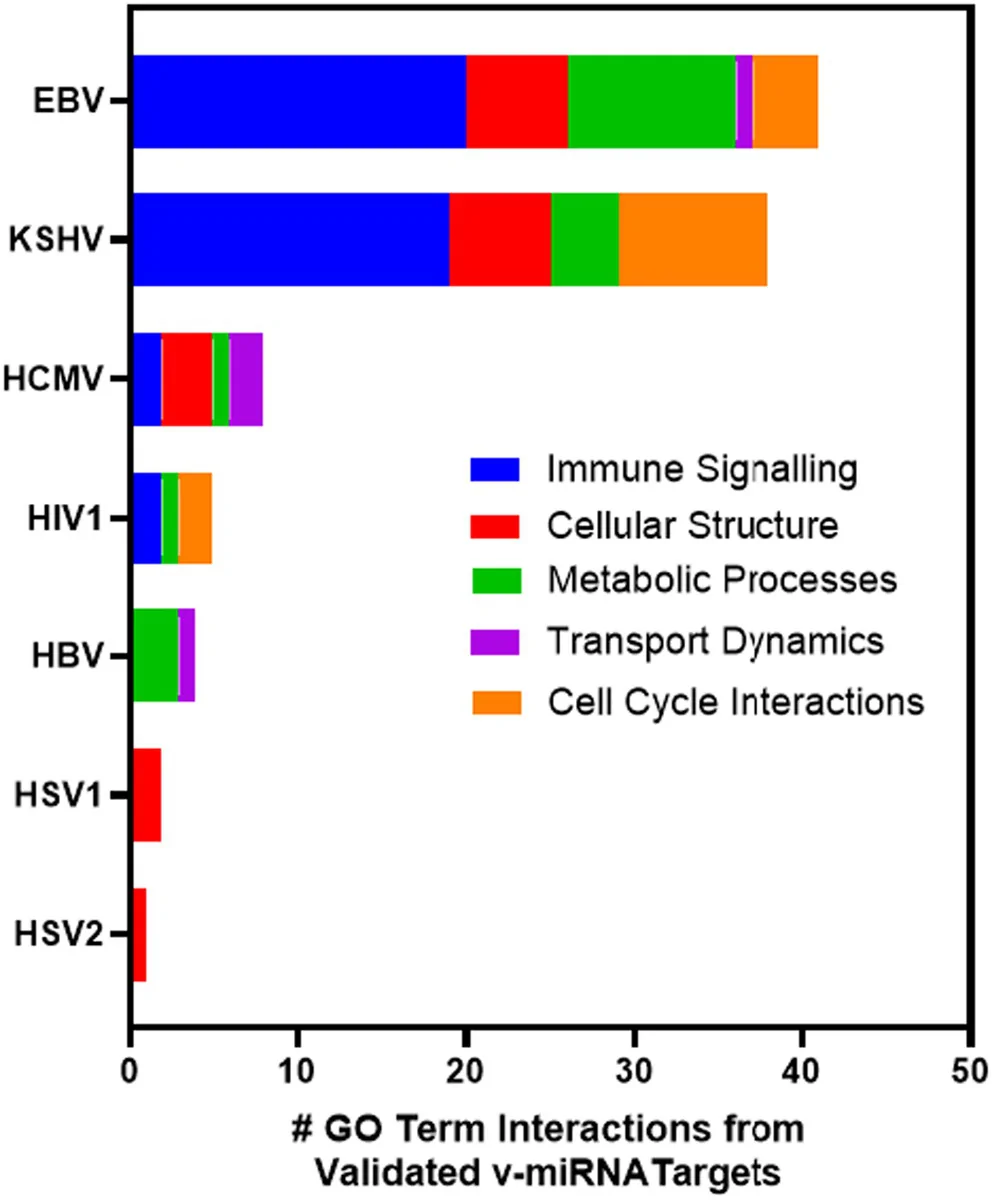
v‐miRNA regulate many biological processes. Human viruses expressing v‐miRNA were collated to identify commonalities in their function. Validated gene targets of each v‐miRNA were analyzed using g‐profiler to identify gene ontology (GO) terms enriched among validated targets. The total number of GO term interactions of v‐miRNA‐expressing viruses, and how they are split among five immunological categories, is displayed.

### 1.2 V‐miRNA Target Host and Viral Genes to Promote Infection

Identifying targets of v‐miRNA has revealed both direct and indirect mechanisms by which they modulate viral infection in humans. Direct promotion of viral persistence is exemplified by EBV BART cluster v‐miRNAs. EBV‐miR‐BART1 suppresses IL12B expression, thereby impairing IL‐12‐dependent priming and effector function of cytotoxic CD8^+^ T cells, a pathway central to antiviral immune surveillance. In the human host, this v‐miRNA‐mediated attenuation of Th1 and CD8^+^ T‐cell responses is thought to facilitate immune evasion during EBV latency and may contribute to sustained viral carriage and EBV‐associated pathology [18]. Given the central role of IL‐12 signaling in antiviral immunity, dysregulation of this by EBV v‐miRNAs may also influence host susceptibility to EBV reactivation and modulate immune control in EBV‐associated malignancies, particularly in immunocompromised individuals.

Another member of this family, BART20, suppresses IFNγ and the pro‐apoptotic factor BAD (Bcl‐2‐associated death promoter), facilitating viral replication and delaying programmed cell death [19, 20]. Similarly, HIV‐1‐derived v‐miR‐TAR‐3p targets multiple host genes regulating apoptosis and cell survival, including Caspase 8 and nucleophosmin, creating a cellular environment that promotes viral persistence and dissemination [21]. By modulating these key host pathways, both EBV and HIV v‐miRNAs can undermine antiviral immune responses and prolong infection in the human host, with potential implications for disease progression and clinical outcomes.

Further along these lines, many polyomaviruses enhance their own survival by using v‐miRNAs to indirectly modulate host genes. The beta polyomaviruses BK virus (BKV), JC virus (JCV), and Merkel cell virus (MKV) each encode a mature v‐miRNA [22, 23, 24]. Interestingly, these v‐miRNA sequences are closely related, and all directly target the viral large T‐antigen [22]. By downregulating T‐antigen expression, these viruses modulate their own replication kinetics and the transition between early and late gene expression [25]. This strategy may contribute to viral persistence and immune evasion within the host. This regulation may contribute to disease pathogenesis, including BKV‐associated nephropathy, progressive multifocal leukoencephalopathy from JCV, and Merkel cell carcinoma, although direct casual evidence remains limited.

Many v‐miRNAs expressed by herpesviruses also target their own genes, contributing to the control of latency and reactivation. HSV‐1 encodes the v‐miRNA v‐miR‐H2‐3p, which represses the transcription and production of the viral‐infected cell protein (ICP‐0), a ubiquitin ligase that promotes viral replication and facilitates reactivation from latency [26]. Viral latency is a critical survival strategy for herpesviruses, enabling the persistent infection of host cells, including neurons, epithelial cells, and immune cells, while simultaneously evading immune surveillance. By modulating ICP‐0 expression, v‐miR‐H2‐3p may influence the frequency of viral reactivation and, consequently, the risk of recurrent disease in infected individuals. HSV‐2 encodes three related v‐miRNAs, v‐miR‐H2, v‐miR‐H3, and v‐miR‐H4, that target ICP‐0 and ICP‐34.5 to further modulate reactivation dynamics [27]. By fine‐tuning the balance between latency and reactivation, these v‐miRNAs enable long‐term viral persistence in the human host, contributing to recurrent genital herpes and influencing disease severity, particularly in immunocompromised individuals.

## 2 Prominent Human Viruses and Their v‐miRNA

### 2.1 Herpesviruses

Herpesviruses are one of the most extensively studied viral families in the context of v‐microRNA. This is attributed to their significant genome size, physical shape, molecular weight, and unique latent/lytic lifecycle, which provides a useful model for researchers to investigate how v‐miRNA regulates complex host–pathogen interactions.

Initially, miRNA was thought to be exclusive to eukaryotes. The discovery of v‐miRNA expression in herpesviruses changed that. Between the Alpha, Beta, and Gamma strain herpesviruses, 141 different mature v‐miRNAs have been identified. In the Alpha group, HSV‐1 and HSV‐2 encode 27 and 24 v‐miRNAs, respectively, while human cytomegalovirus (HCMV) is the only Beta group virus discovered thus far to encode v‐miRNA and has 21 [21, 28]. The gamma‐herpesvirus, EBV, encodes 44 mature v‐miRNAs and Kaposi's sarcoma‐associated herpesvirus encodes 25 [2, 29]. While the specific mechanisms of action of all herpesvirus v‐miRNAs remain to be confirmed, preliminary evidence indicates that they primarily regulate genes, both host and viral, responsible for the establishment and maintenance of latency [26].

Multiple HSV v‐miRNAs, including hsv‐miR‐H2, H3, H4, and H6, target ICP‐0, ICP‐4, and ICP‐34.5, demonstrating autoregulatory roles in viral replication and latency [26, 27]. Beyond viral transcripts, some HSV v‐miRNAs also modulate host gene expression. For example, HSV‐1 miR‐H27 targets the cellular transcriptional repressor Kelch‐like 24 (KLHL24), thereby influencing pathways involved in immune evasion, viral replication, and cell proliferation [30]. Through the coordinated regulation of both viral and host genes, HSC v‐miRNAs may contribute to the establishment and maintenance of latency, regulation of viral reactivation, and influence the severity of the infection in humans.

EBV v‐miRNA are organized into three genomic clusters, BHRF1, BART1, and BART2 [31, 32]. The BHRF1 cluster, the smallest of the three, includes ebv‐miR‐BHRF1‐3p, which has been implicated in suppressing cytokine production to promote immune evasion [33]. One validated target of EBV‐miR‐BHRF1‐3p is TAP2 (Figure 3), a critical component of the transporter associated with antigen processing (TAP) complex. TAP2 forms part of the machinery responsible for loading peptides into MHC Class 1 molecules for presentation to CD8^+^ T cells. By reducing TAP2 expression and impairing antigen presentation, EBV‐miR‐BHRF1‐3p may decrease CD8^+^ T‐cell recognition of infected cells, thereby promoting viral persistence and contributing to EBV‐associated pathologies in the human host.

**FIGURE 3:**
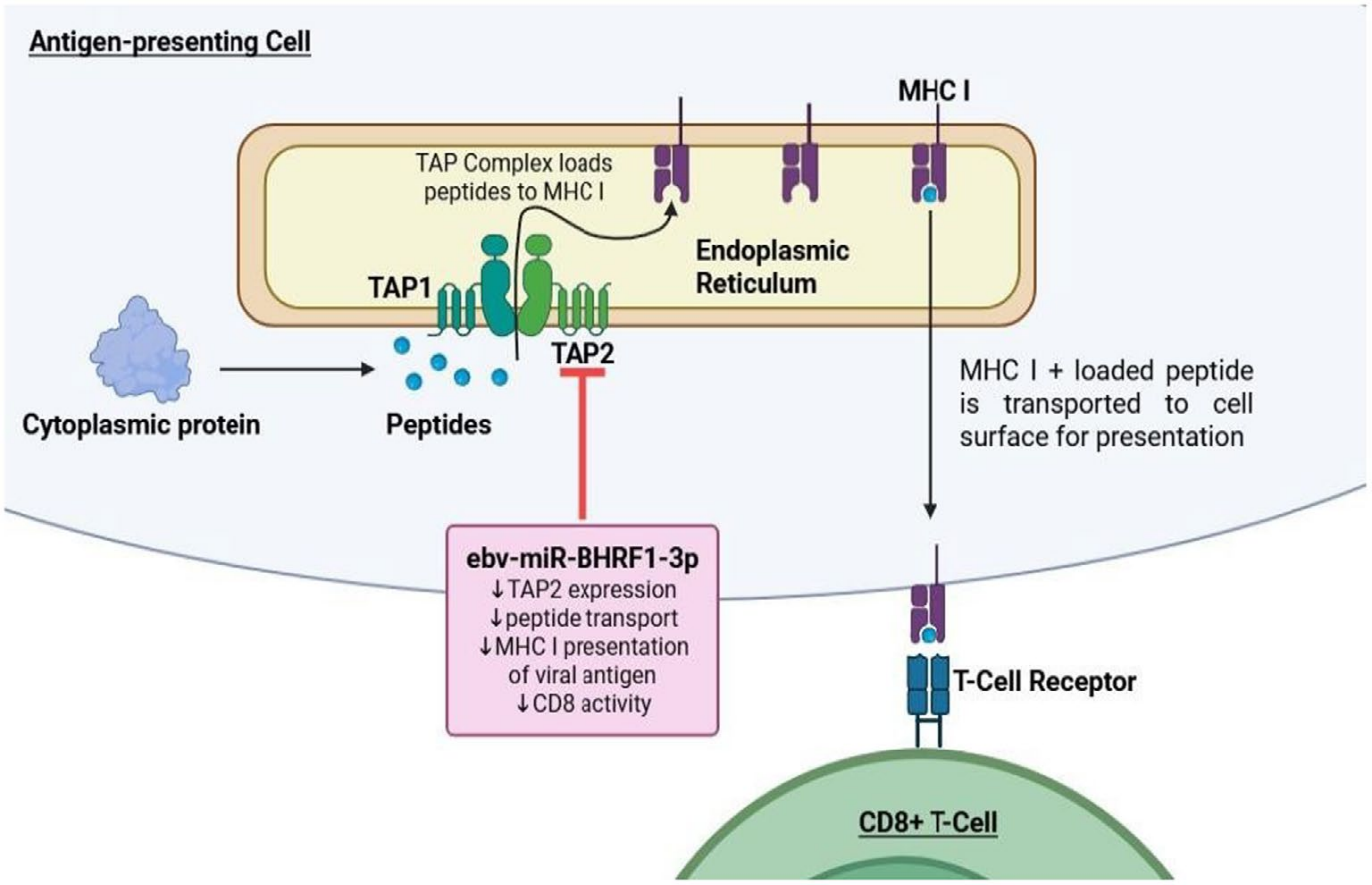
T‐cell activation is inhibited in humans through TAP2 inhibition by EBV miR‐BHRF1‐3p. Transporter associated with antigen processing 2 (TAP2) is a validated target of the EBV‐encoded v‐miR‐BHRF1‐3p. TAP2 and TAP1 form the TAP complex, responsible for the transport of peptides from the cytoplasm into the endoplasmic reticulum and onto MHC Class I molecules. EBV miR‐BHRF1‐3p downregulates the expression of TAP2 so fewer TAP complexes can be formed. This reduces transport of viral antigen peptides to MHC I molecules, leading to reduced CD8^+^ T‐cell recognition and activation.

The BART clusters in EBV encode several v‐miRNAs that promote viral survival and replication by modulating host immune responses. For example, EBV‐miR‐BART16‐3p and EBV‐miR‐BART17‐5p downregulate the viral oncogene latent membrane protein 1 (LMP1) [34]. LMP1 is a central regulator of TNF receptor‐associated signaling pathways, including MAPK, PI3K/Akt, and NF‐κB, all of which are critical for immune activation and antiviral responses. By dampening LMP1 expression, these v‐miRNAs help EBV evade immune detection and may contribute to persistent infection and oncogenic transformation in humans.

Other EBV‐miRNA, such as EBV‐miR‐BART21 and EBV‐miR‐BART22, promote immune evasion by targeting latent membrane protein 2A (LMP2A), a viral antigen recognized by cytotoxic T cells [35]. BART cluster miRNAs may also modulate host cell survival and the cell cycle; for instance, miR‐BART5 suppresses the pro‐apoptotic factor PUMA, enhancing infected cell survival and promoting the establishment and maintenance of viral latency [36]. Collectively, these EBV v‐miRNAs can help the virus persist in the human host while evading immune detection, laying the groundwork for long‐term infection and potential disease progression.

### 2.2 Human Immunodeficiency Virus 1

Few v‐miRNA have been identified in RNA viruses. HIV‐1 is the only human‐infecting RNA virus listed in the miRBase v22. As a retrovirus, HIV‐1 undergoes reverse transcription of its RNA into DNA, which is subsequently integrated into the host genome within the nucleus. This integration enables the host genome to transcribe the viral genetic material, including potential v‐miRNAs. Initial in silico prediction analysis indicated that HIV‐1 encodes five putative pre‐miRNAs, while further experimental validation has confirmed the presence of several HIV1‐v‐miRNAs [37].

It should be noted that, for many HIV‐1 v‐miRNAs, there is ongoing discussion regarding their classification as miRNAs. Some HIV‐1 miRNAs are derived from focused areas in their genome, unlike human miRNAs, which are more dispersed. Also, HIV‐1 v‐miRNAs are often < 20 nucleotides, despite miRNAs generally being accepted as 20–24 nucleotides long [37]. Furthermore, while their pri‐miRNA form does include a stem loop, it lacks some of the defining features of generally accepted pri‐miRNA stem‐loops, such as Drosha recognition motifs or a more flexible structure [37]. Some studies have suggested that HIV‐1 does not encode any short‐interference RNA (siRNAs) or miRNAs. Varied studies have collected evidence from HIV‐1‐infected T cells and primary peripheral blood mononuclear cells (PBMCs) that support this claim [38, 39].

The arguments against the existence of HIV‐1 miRNAs are primarily based on both technical and biological concerns. Some researchers contend that the small RNA fragments detected as miRNAs are, in fact, degradation products or noncanonical small noncoding RNAs that do not undergo classical miRNA biogenesis and therefore should not be classified as true miRNAs [40] and have offered alternative explanations for the changes observed in these studies. It has been suggested that these effects result from RNA degradation fragments interfering with host protein machinery or artifacts arising from plasmid‐based overexpression systems [40]. Others argue that altered viral replication and host gene expression may instead be mediated by HIV‐1 RNAs and proteins, which can modulate interferon signaling, stress responses, and other aspects of the host immune system, rather than by bona fide viral miRNAs [39]. Highlighting the persistence of this debate, a 2024 study predicted the existence of HIV1 v‐miRNA using bioinformatics and deep sequencing tools before experimentally validating their work with RT‐PCR and microarrays. Notably, while the identified sequences met several of the classical miRNA criteria, their functional relevance was demonstrated using plasmid‐based overexpression, the very system that skeptics caution can introduce experimental artifacts. Nevertheless, the overexpression sequences altered viral replication [41], ensuring that whether these short RNA fragments are “true” miRNA or functional degradation products remains a central topic of discussion, and research to investigate the marked effect of small RNAs in HIV1 viral replication continues.

Despite the ongoing debate regarding their biogenesis, several putative HIV‐1 v‐miRNAs have identified functional roles in modulating both viral and host factors to favor viral persistence. One of these, HIV1‐v‐miR‐N367, suppresses the viral protein, negative regulatory factor (Nef). Nef enhances HIV infectivity by promoting viral replication and host cell apoptosis (Figure 4) [42]. Suppression of Nef by HIV‐1‐v‐miR‐N367 may be a viral strategy to moderate immune system activation, to prevent clearance of infected cells, and thereby promote long‐term persistence. This idea is supported by two studies that discovered hiv1‐miR‐TAR‐3p and hiv1‐miR‐TAR‐5p [43, 44]. These miRNAs target host apoptotic regulators, including the excision repair cross‐complementation Group 1 (ERCC1) gene and the immediate early response 3 (IER3) gene, which are both involved in DNA repair and apoptosis inhibition. This may prolong the survival of infected cells, potentially extending the time for viral replication to occur. HIV‐1 v‐miRNA may also modulate host cytokine responses. V‐miR‐88 and v‐miR‐99 have been shown to trigger TNF release by macrophages, potentially influencing systemic immune activation [45]. Together, these observations highlight the multifaceted role of HIV v‐miRNAs in balancing viral replication, host cell survival, and immune modulation to facilitate their persistent infection in humans.

**FIGURE 4:**
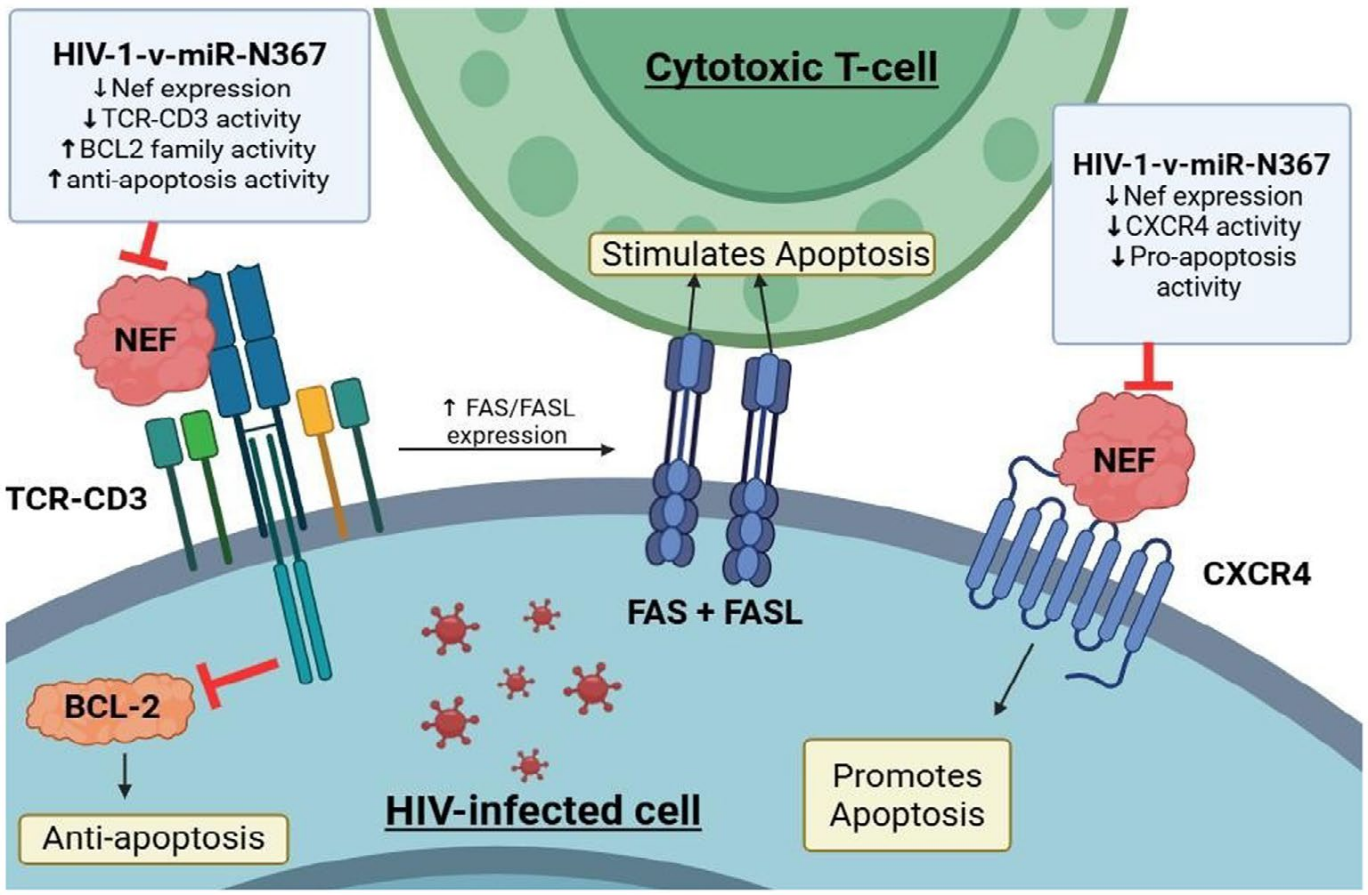
Downregulation of viral protein Nef by HIV‐1 v‐miR‐N367 regulates latency. HIV‐1‐v‐miR‐N367 targets the Nef‐coding gene, reducing its production during viral infection. Nef can enhance HIV spread by activating apoptosis through CXCR4 and by binding to the TCR‐CD3 receptor to reduce BCL‐2. Nef also increases FAS/FASL expression to stimulate cytotoxic T‐cell‐induced apoptosis. Nef's inhibition by HIV‐1‐v‐miR‐N367 therefore reduces proapoptotic pathways and increases antiapoptosis pathways. This may contribute to persistence of infected cells and influence viral replication dynamics.

### 2.3 Influenza

Influenza virus (IAV) is among the most extensively studied RNA viruses. Typically, RNA viruses are not considered capable of miRNA production as they reproduce in the cytoplasm of their host, which precludes their access to the nuclear protein Drosha, essential for processing pri‐RNAs into pre‐miRNA [46].

However, IAV is unlike most RNA viruses and replicates in the nucleus of infected cells, allowing it to overcome this barrier. A study from 2018 utilized deep sequencing of influenza virus‐infected cells to identify the presence of viral miRNA‐like molecules in H5N1 [47]. The highly pathogenic influenza strain encoded a miRNA‐like molecule, v‐miR‐HA‐3p, and was shown to exacerbate the cytokine storm characteristic of severe infection. V‐miR‐HA‐3p suppresses poly(rC)‐binding protein 2 (PCBP2), which inhibits the pattern recognition receptor RIG‐I and MAVS protein to reduce downstream activation of TNF receptor‐associated factors (TRAFs) (Figure 5). These TRAFs are responsible for upregulating of pro‐inflammatory cytokines and Type I Interferons via NF‐κB and IRF3/7. PCBP2's role is therefore to limit the expression of these markers such that they do not cause a cytokine storm. Cytokine storms are a hallmark of severe IAV infections and can lead to further complications such as acute respiratory distress syndrome (ARDS). By reducing PCBP2 expression, IAV‐v‐miR‐HA‐3p causes IAV‐infected cells to overproduce cytokines and IFNα and β, leading to the formation of a cytokine storm in infected persons. Inhibition of v‐miR‐HA‐3p, using antagomirs, significantly reduced viral replication and improved survival in infected mice [47], highlighting its potential as a target for therapeutic intervention, to mitigate hyperinflammation and improve clinical outcomes in severe influenza.

**FIGURE 5:**
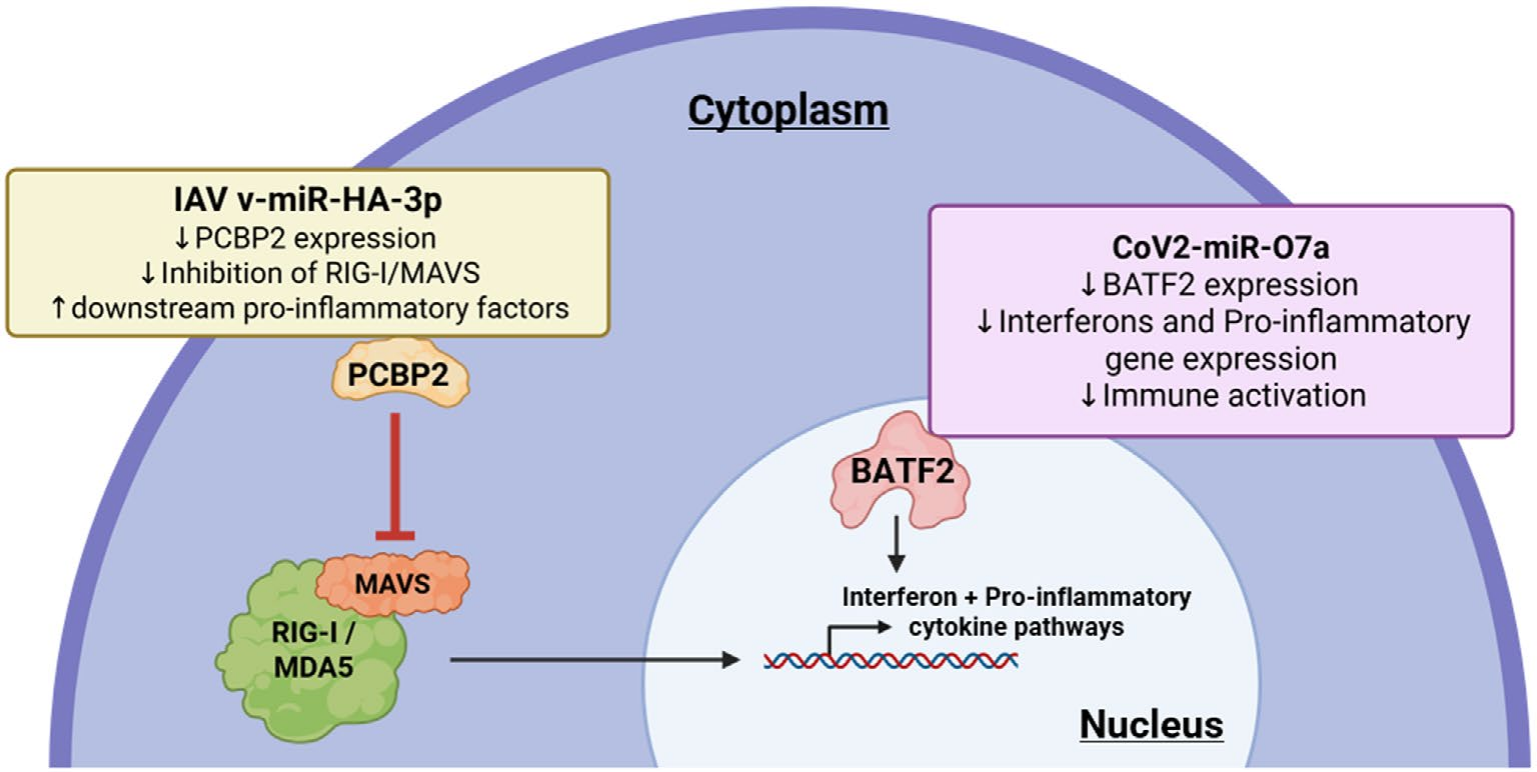
IAV and Coronavirus‐encoded miRNA interfere with pro‐inflammatory gene expression. Poly(rC)‐binding protein 2 (PCBP2) is a validated target of the IAV miRNA, IAV‐v‐miR‐HA‐3p. Normally, PCBP2 acts as a negative regulator of RIG/MAVS‐mediated antiviral immunity, blocking hyperactivation of the immune system that can lead to cytokine storms. By suppressing PCBP2 expression, v‐miR‐HA‐3p removes this block, resulting in overinduction of Type I Interferons and pro‐inflammatory genes and cytokines. Conversely, Coronavirus‐encoded miRNA CoV2‐miR‐O7a reduces inflammation to aid infection establishment. It can downregulate basic leucine zipper ATF‐like transcription factor 2 (BATF2), responsible for the induction of pro‐inflammatory mediators such as NOS2, TNF, CCL5, IL‐12b, and SOCS1. Diminishing IFN‐stimulated gene output and weakening effector cell differentiation and antiviral responses may aid infection establishment.

Along with its innate ability to produce v‐miRNA, IAVs unique replication cycle has also been used as a vector for delivering exogenous miRNA. In one study, the insertion of a human pri‐miRNA sequence for miR‐124 into the IAV genome enabled the modified virus to successfully produce functional miR‐124 in host cells without impacting viral replication [48]. This strategy is dependent on IAV's nuclear localization, with the virus trafficking to the nucleus, the engineered transcript gaining access to host Drosha machinery required to cleave the pri‐miRNA construct. The approach holds promise for the development of attenuated miRNA‐targeted IAV‐based vaccines.

### 2.4 Coronaviruses

Coronaviruses, including SARS and SARS‐CoV‐2, are cytoplasmic single‐stranded RNA viruses that replicate exclusively in the cytoplasm. This presents a challenge to the traditional paradigm of v‐miRNA biogenesis, which relies on the Drosha‐Dicer pathway for processing and producing the mature miRNA molecule. Unlike nuclear‐replicating IAV, coronaviruses remain in the cytoplasm and must therefore utilize noncanonical processing pathways to generate v‐miRNA.

The strongest evidence supporting coronavirus‐encoded miRNAs comes from the identification of CoV2‐miR‐O7a [49]. This small RNA exhibits the hallmark characteristics of a v‐miRNA, measuring 20–25 nucleotides in length and deriving from a hairpin structure in the ORF7a region of SARS‐CoV‐2. Pawlica et al. demonstrated that CoV‐miR‐O7a associates with host argonaute loading proteins, which are essential for the execution of micro‐RNA‐mediated RNA silencing complexes. Notably, although CoV‐miR‐O7a relies on host cell machinery, the biogenesis bypasses the canonical Drosha‐dependent nuclear cleavage step.

Functional studies demonstrated that CoV2‐miR‐O7a downregulates two critical host mRNA targets: basic leucine zipper ATF‐like transcription factor 2 (BATF2) and heparan sulfate proteoglycan 2 (HSPG2). BATF2 is an interferon‐inducible transcription factor that promotes antiviral gene expression and supports cytotoxic lymphocyte differentiation (Figure 5), whereas HSPG2 acts as a structural and signaling proteoglycan, influencing leukocyte trafficking and tissue repair. The targeting of these two factors may provide a mechanism whereby SARS‐CoV‐2‐derived miRNAs, like many other miRNA‐encoding viruses, modulates host pathways important for infection establishment.

The identification of other coronavirus‐encoded small RNAs predates the SARS‐CoV‐2 pandemic. In 2017, three small‐viral‐derived miRNAs—svRNA‐N, svRNA‐nsp3.1, and svRNA‐nsp3.2—were identified in coronavirus‐infected lung tissue and cell cultures. Luciferase reporter assays in DBT‐mACE2 cells, a murine brain‐derived cell line engineered to express human ACE2, demonstrated that these molecules can suppress host gene expression, hinting at potential regulatory capabilities, though further studies are needed to confirm precise targets and whether this function suggests their possible classification as v‐miRNA [50].

Recent literature, using bioinformatics and deep‐learning frameworks to predict putative SARS‐CoV‐2 v‐miRNA, identified a further 27 potential candidates. Research to confirm and understand how these putative v‐miRNAs may regulate COVID‐19 infection is ongoing [51]. While still largely speculative, these computational prediction models have revealed putative sequences conserved across 80 different SARS‐CoV‐2 genomes that are predicted to target host genes involved in immune regulation, apoptosis, and viral entry [52, 53, 54]. Predicted targets include PDK1, a regulator of Akt signaling and cell survival [55], as well as molecules involved in TGFβ and TMPRSS2 expression. Most of these proposed interactions are based on bioinformatic analysis and require further experimental validation. Consequently, while these studies raise the possibility that SARS‐CoV‐2 derived v‐miRNAs may influence multiple host cellular pathways during infection, their biological significance in Coronavirus pathogenesis remains to be verified.

### 2.5 Ebola

Although Ebola virus (EBOV) is a nonsegmented negative‐sense RNA filovirus and therefore not traditionally expected to encode microRNAs, multiple independent studies have demonstrated that EBOV produces a diverse repertoire of functional v‐miRNAs during infection [9, 56, 57, 58]. These miRNAs have been detected in vitro, in animal models, and in human clinical samples.

One of the most clearly defined EBOV v‐miRNA–target interaction involves Zebov‐miR‐1‐5p, a viral miRNA encoded within the 5′ UTR of EBOV viral protein 24 [57]. Zebov‐miR‐1‐5p is structurally and functionally similar to human miR‐155‐5p, a key regulator of inflammatory signaling [59]. Luciferase and western blot assays validated targets of Zebov‐miR‐1‐5p. Like miR‐155‐5p, Zebov‐miR‐1‐5p directly repressed importin‐α5 (KPNA1), the nuclear transport adaptor required for STAT1 translocation following Type I and II IFN stimulation. KPNA1 downregulation prevents STAT1 from entering the nucleus, thereby blocking transcription of interferon‐stimulated genes (Figure 6).

**FIGURE 6:**
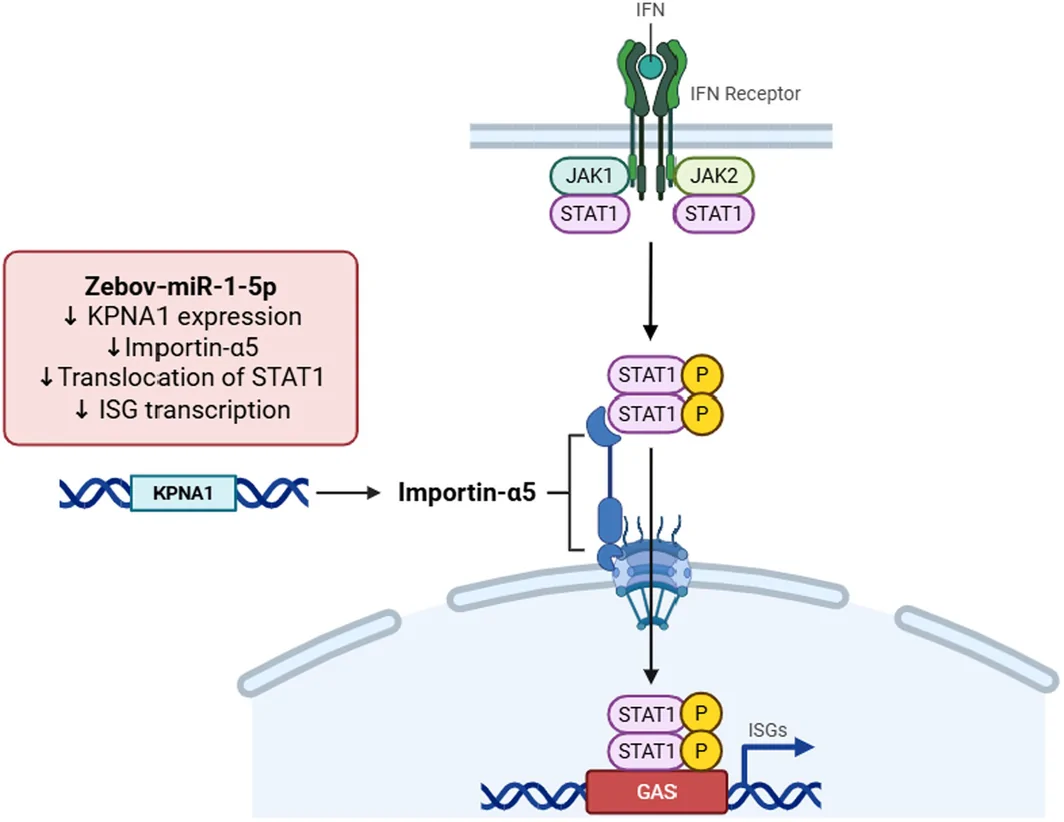
Zebov‑miR‑1‑5p‐mediated inhibition of interferon signaling through KPNA1 repression. Ebola virus‑encoded miRNA Zebov‑miR‑1‑5p suppresses host antiviral signaling by targeting KPNA1, which encodes importin‑α5, the adaptor required for nuclear translocation of phosphorylated STAT1 following IFN stimulation. Reduced KPNA1 expression limits importin‑α5 availability, preventing STAT1 dimers from entering the nucleus and binding to GAS elements to drive transcription of interferon‑stimulated genes (ISGs). This miRNA‑mediated blockade complements EBOV VP24 protein‑driven inhibition of STAT1, creating a multilayered approach to reduce host interferon responses.

This single interaction may have important immunological consequences. EBOV is already known to inhibit STAT1 through VP24 protein‐mediated sequestration of host proteins like KPNA1, thereby impairing STAT1 nuclear translocation [60]. The addition of a miRNA‐mediated mechanism targeting KPNA1 suggests EBOV may employ multiple strategies to reduce IFN signaling and further limit STAT1‐dependent antiviral responses. Functionally, this may contribute to the high viral replication that occurs before adaptive responses can be mobilized. The observed miR‐155 mimicry is consistent with the possibility that EBOV exploits host inflammatory regulatory pathways, although the biological significance of this interaction remains to be fully defined.

Other EBOV miRNAs were identified in genome‐wide target prediction and network analysis during the 2014 Ebola outbreak include the T‐series (miR‐T1‐3p/5p, miR‐T2‐3p/5p, miR‐T3‐3p/5p, miR‐T4‐3p) [58]. Among the 138 predicted human targets of these, two important immune regulators stand out: NFKBIE (IκBε) and RIPK1, key modulators of NF‐κB activation and TNF signaling, respectively. Experimental validation in Hela cells transfected with synthetic mimics demonstrated that EBOV‐miRNAs T1‐5p, T3‐5p, and T4‐3p reduced NFKIBIE mRNA expression while all T‐series EBOV‐miRNA reduced RIPK1 expression. Collectively these findings suggest that EBOV miRNAs may influence both NF‐κB‐ and TNF‐dependent pathways, two critical regulators of antiviral immunity and inflammation. By targeting upstream signaling molecules rather than individual cytokines themselves, EBOV miRNAs may contribute to broad alterations in host immune responses and inflammation during infection, although their precise role in EBOV immunopathology remains to be fully defined.

## 3 Conclusion

MiRNA have emerged as pivotal regulators of gene expression and host immunity. The awarding of the 2024 Nobel Prize for Physiology or Medicine for the discovery of miRNA underscores this impact. MiRNA fundamentally transformed the way scientists viewed “junk genes” and our understanding of how our bodies regulate gene expression. Despite being identified almost 30 years ago in humans, we still know comparatively little about their roles in infection. Viral microRNA only adds to this complexity. V‐miRNAs' interaction with both self and host genes expands the possibilities for how viruses facilitate infections, evade host immune responses, and establish latency. Are v‐miRNA targeting the host mRNA to suppress antigen presentation, or are v‐miRNA competing with the host miRNA for binding sites, or maybe, it is the v‐miRNA acting on its own genes to regulate viral replication or delay apoptosis? These scenarios are not mutually exclusive and may operate concurrently during infection. Research on the expression of v‐miRNA, as well as their targets, is a slow process. Further research into v‐miRNA will undoubtedly improve our understanding of viruses and the mechanisms they employ to infect and spread among humans. This improved understanding may lead to the development of new safe and effective antiviral therapies.

## Author Contributions

**Bernadette M. Saunders:** supervision, conceptualization, funding acquisition, writing – review and editing, project administration. **Riley J. K. Easton:** conceptualization, investigation, writing – original draft, validation, visualization, writing – review and editing, data curation.

## Funding

This work was supported by the National Health and Medical Research Council (Grant no. 2027815) and Australian Government Research Training Program.

## Conflicts of Interest

The authors declare no conflicts of interest.

## Data Availability

The data that support the findings of this study are available in public repositories. A range of databases and software were used for the purpose of writing this review and designing figures. These were: miRbase (https://www.mirbase.org/), DIANA‐MicroT (https://dianalab.e‐ce.uth.gr/microt_webserver/#/interactions), VIRmiRNA (http://crdd.osdd.net/servers/virmirna), and g‐profiler (https://biit.cs.ut.ee/gprofiler/gost).
